# Violence and Abuse: A Pandemic Within a Pandemic

**DOI:** 10.5811/westjem.58405

**Published:** 2023-07-17

**Authors:** Paula J. Whiteman, Wendy L. Macias-Konstantopoulos, Pryanka Relan, Anita Knopov, Megan L. Ranney, Ralph J. Riviello

**Affiliations:** 1 Cedars-Sinai Medical Center, Los Angeles, California; 2 Center for Social Justice and Health Equity, Boston, Massachusetts; 3 MGH Freedom Clinic, Boston, Massachusetts; 4 Harvard Medical School, Department of Emergency Medicine, Boston, Massachusetts; 5 Massachusetts General Hospital, Department of Emergency Medicine, Boston, Massachusetts; 6 Emory Healthcare Network, Atlanta, Georgia; 7 World Health Organization, Emergency, Trauma and Acute Care Programme, Geneva, Switzerland; 8 Brown University, Warren Alpert Medical School, Providence, Rhode Island; 9 Yale School of Public Health, Yale University, New Haven, Connecticutt; 10 University of Texas Health San Antonio, Lozano Long School of Medicine, Department of Emergency Medicine, San Antonio, Texas

**Keywords:** pandemic, elder abuse, human trafficking, COVID-19, gun violence, intimate partner violence, child abuse, fear

## Abstract

**Introduction:**

During the COVID-19 pandemic, as society struggled with increasing disease burden, economic hardships, and with disease morbidity and mortality, governments and institutions began implementing stay-at-home or shelter-in-place orders to help stop the spread of the virus. Although well-intentioned, one unintended adverse consequence was an increase in violence, abuse, and neglect.

**Methods:**

We reviewed the literature on the effect the pandemic had on domestic violence, child and elder abuse and neglect, human trafficking, and gun violence. In this paper we explore common themes and causes of this violence and offer suggestions to help mitigate risk during ongoing and future pandemics. Just as these forms of violence primarily target at-risk, vulnerable populations, so did pandemic-related violence target marginalized populations including women, children, Blacks, and those with lower socioeconomic status. This became, and remains, a public health crisis within a crisis. In early 2021, the American College of Emergency Physicians (ACEP) Public Health and Injury Committee was tasked with reviewing the impact the pandemic had on violence and abuse as the result of a resolution passed at the 2020 ACEP Council meeting.

**Conclusion:**

Measures meant to help control the spread of the COVID-19 pandemic had many unintended consequences and placed people at risk for violence. Emergency departments (ED), although stressed and strained during the pandemic, remain a safety net for survivors of violence. As we move out of this pandemic, hospitals and EDs need to focus on steps that can be taken to ensure they preserve and expand their ability to assist victims should another pandemic or global health crisis develop.

## INTRODUCTION

In early 2020, as the world was thrust into the COVID-19 pandemic, countries struggled with increasing disease burden, economic hardships, and disease morbidity and mortality, which led to the implementation of stay-at-home orders to help stop viral spread. This led to increased stress, anxiety, and work/school absence.^1^ Unintended adverse consequences included increases in violence, domestic violence (DV), child and elder abuse and neglect, human trafficking, and gun violence. In this article we look at the impact of the COVID-19 pandemic on violence and its relationship to DV, child and elder abuse and neglect, human trafficking, and gun violence, and we offer suggestions to help mitigate violence and better manage our response in the face of this uncertainty.

## METHODS

A group of experts in the topics of DV, child abuse and neglect, human trafficking, elder abuse and neglect, and gun violence came together to summarize the literature available regarding the COVID-19 pandemic and its impact on these topics.

## DOMESTIC VIOLENCE

Soon after the implementation of pandemic mitigation measures, reports of DV surged globally. This led to United Nations Secretary Guterres’ ominous warning: “We know lockdowns and quarantines are essential to suppressing COVID-19, but they can trap women with abusive partners . . . Over the past weeks, as the economic and social pressures have grown, we have seen a horrifying surge in domestic violence.”^2^ Media reports quickly called attention to links between pandemic lockdown orders and worldwide increases in intimate partner violence.^3^ While anyone can be a victim of DV, women are disproportionately affected; thus, for this paper we refer to female victims.

Researchers in New Zealand previously showed that all forms of family violence (DV, child abuse, and elder abuse) increase during and after large-scale crises.^4^ Examples of the widespread impact of pandemic lockdowns are abundant. In 2020 the *Guardian* reported a global surge in reports of DV.^5^ Brazil experienced a 40–50% increase in DV, and Spain had a 20% increase in the number of helpline calls in the first few days of lockdown.^5^ In Cyprus, the number of hotline calls rose 30% within one week of its first COVID-19 case.^6^ In the United Kingdom (UK), Refuge—one of the leading domestic abuse organizations—reported a 25% increase in helpline calls in the seven days following UK lockdown measures.^6^ During the same period, Refuge noted a 150% increase in website visits.^7^ In China’s Hubei province, DV tripled when comparing February 2020 to February 2019.^6^ In France reports of DV increased 30% and in Argentina 25%.^6^

In March 2020 reports of DV within the United Sates followed a similar trajectory: the Portland [Oregon] Police Bureau recorded a 22% increase in family violence calls^8^; the San Antonio [Texas] Police Department saw an 18% increase^9^; in Alabama the Jefferson County Sheriff’s Office reported a 27% increase in March 2020 compared to March 2019^11^; and the New York City Police Department responded to a 10% increase in DV calls in March 2020 compared to March 2019.^11^ In February 2021, the National Commission on COVID-19 and Criminal Justice (NCCCJ) reported that DV incidents in the US increased by 8.1% after lockdown orders were issued.^12^ The NCCCJ report included police call logs, DV crime reports, emergency line registries, and health records. Despite these increases in reports and hotline calls, US emergency departments (ED) saw a significant decrease in visits related to intimate partner violence (442 vs 484) and suspected child abuse and neglect (884 vs. 1,038) during March 15-October 10, 2020, compared to the same period in 2019.^13^

Hotline and helpline calls surged in the US, with the National DV Hotline reporting a contact volume increase of 9% and ≈10% of callers citing COVID-19 as a factor.^15^ Between March-May 2020, 90% of callers reported experiencing emotional/verbal abuse, 61% physical abuse, 16% digital abuse (use of technology to bully, harass, stalk, or intimidate), and 11% sexual abuse.^15^ Some hotlines noted decreased call volumes as survivors were unable to access hotlines due to isolation and abuser contact.

Homicides related to DV increased. In 2020 more than 2,000 people were killed in the US in DV-related shootings, an increase of 4% from 2019, with disproportionate increases in Texas (69%), Maryland (93%), Missouri (67%), and Utah (160%).^16^ In a survey of law enforcement personnel focused on DV response, 33% reported an increase in DV homicides in their communities and half reported abusers threatening to shoot survivors.^17^ Spain’s first DV fatality occurred five days after lockdown. The UK as well saw an increase in DV-related homicides.^18^

Cases of DV rose during the pandemic as lockdown placed those vulnerable populations in close proximity to their abusers.^19^–^22^ Social isolation of survivors made them more susceptible to abuse with few resources for help. Unemployment, economic/financial strain, disease fears, childcare stress and homeschooling, depression, and drug and alcohol use all increased DV risk in the home, resulting in an increase in all forms of violence. Those victims in pre-existing violent relationships as well as in previously non-violent relationships had difficulty reaching out to DV hotlines; while some hotlines had dramatic increases in call volume, others experienced fewer calls. Without in-person access to family, friends, and co-workers, visible injuries go unnoticed and subtle clues may have been missed with face masks hiding visible facial trauma. Video-conferencing platforms allow cameras to be off or adjusted, blocking physical signs of the abuse or the abuser off-screen.

Aid from social service agencies, DV agencies, shelters, and rape crisis centers was limited with some organizations deemed non-essential. Infrastructure, technology, and financial limitations curtailed the transition to remote response. Remotely staffed hotlines and helplines stayed open. Shelters faced losses of volunteers and workers and difficulty implementing social distancing and personal protective equipment (PPE) protocols and cleaning/disinfecting measures in the face of supply shortages.

Many EDs and hospitals severely restricted visitors, including DV and sexual assault advocates, crisis workers, and shelter staff, leaving victims without adequate support while being evaluated for injuries or following sexual assault. When allowed into the ED or hospital, agencies were required to provide their own PPE, despite supply shortages. Going forward, hospitals should establish policies allowing social service agencies access to survivors and to provide those workers with appropriate PPE during a pandemic. Emergency departments need to ensure they screen ALL patients for violence at the time of the visit/hospitalization and provide appropriate agency referrals. Given the unprecedented access abusers have to victims, resources need to be compact, easily concealable, and non-discoverable. Hospitals should work with local agencies to ensure access to services, personnel, and resources. State governments need to re-classify social service agencies as essential, allowing them to continue their important work.

Agencies need to do the following: 1) develop protocols and policies that allow for easy transition to work from home; 2) enhance information technology infrastructure in anticipation of future pandemics or lockdowns with staff education; and 3) institute routine, camera-on employee checks to ensure their well-being.

## CHILD ABUSE AND NEGLECT

The World Health Organization, United Nations Women, and UNICEF released a joint statement calling for the protection of children from violence including maltreatment, gender-based violence, and sexual exploitation.^23^ A report from the US Centers for Disease Control and Prevention found that despite a dramatic decrease in total pediatric ED visits during lockdown, the number of hospitalizations from child abuse and neglect remained stable, representing a dramatic increase in the yearly percentage of ED visits related to child abuse and neglect among all age groups.^24^ The National Child Abuse Hotline allows anyone, including children, to call in or report. In 2018 and 2019 the hotline received 93,000 and 90,000 calls, respectively.^25^,^26^ By contrast, in fiscal year 2020 there were over 112,000 calls, representing a 23% increase.^27^

The same COVID-19 lockdown measures affecting DV survivors affected children as well. This includes social isolation, virtual education, and financial and housing insecurities. The presence of children at home continuously instead of away at school or daycare led to added stress, with parents and caregivers denied respite from direct childcare duties. Home life became private. Without visitors to the home and children barred from attending school and extracurricular activities, there was no direct interaction with potential, mandated reporters or concerned citizens. Children had less opportunity to privately confide in or ask for help from teachers, counselors, friends, and healthcare personnel who would otherwise recognize signs of abuse.

If an individual doesn’t already live in a safe environment, then lockdown becomes more dangerous to them. Sheltering in place may lead to child neglect as supervising adults engage in other necessary tasks. Abusers having unlimited access to new household members, both related and non-related, in shared living space, potentially placed children at further risk.

Without the in-person supervision of teachers or other school-based mandated reporters, virtual learning limits assessment of children for abuse or neglect, especially as virtual learning via cameras only shows part of the child or their environment. The actual household environment was potentially obscured with preloaded backgrounds or children being outside the home to access better Wi-Fi.

Similarly, case workers conducting virtual visits were not able to fully assess home-life situations. Food insecurity may have been missed. Children who relied on school breakfast and lunch programs as their source of healthy nutritious meals lacked adequate nutritious food during lockdown, negatively affecting health and learning. Mandated reporters did not have the same level of pre-pandemic contact with children, given the implementation of virtual learning and telemedicine visits. Abusers had greater ability to cover up or limit visualization of telltale signs of abuse. Official reports to child protective services decreased significantly by about 20–70%, possibly attributable to fewer in-person contacts with mandated reporters.^27^

Child abuse and neglect is preventable. Pandemic and disasters require heightened methods of surveillance, reporting, and investigation of cases. Prevention strategies include the following: offering economic support; allowing parents flexible work schedules to balance childcare and work responsibilities; and implementing mechanisms to get children safely back in school for their mental health and physical well-being. Schools need flexibility for in-person services for children, including access to nutritious meals with community support to help with these efforts.

A visit to the ED may be a child’s only access to help. Emergency physicians should conduct thorough history and physical exams of children, paying attention to emotional well-being, signs of physical injury, neglect, and other red flags of child abuse. Consults to social services and child protective services (CPS) should not be restricted due to a pandemic or limited access to PPE. The CPS agencies must have mechanisms to continue to conduct in-person and in-hospital evaluations and have processes for virtual home visits with the ability to provide other needed services.

## EXPLOITATION AND HUMAN TRAFFICKING

Societal safety measures meant to protect against COVID-19 transmission further isolated at-risk, exploited, and trafficked individuals, posing added barriers to potential victim identification and assistance. Vulnerability to exploitation and trafficking has been exacerbated by both the rise in family violence and household financial insecurity. Widespread school closures unique to the COVID-19 pandemic resulted in children spending more time online, possibly unsupervised, as parents or legal guardians who were essential workers had to juggle work and homelife.

The remote digital era ushered in by the COVID-19 pandemic led to exponential growth in predatory cyber activity including the targeted solicitation of minors through social media, chat rooms, and gaming platforms. As early as the first quarter of 2020, cybersecurity groups began to detect chatter within child sexual abuse material (CSAM) subscription forums and other parts of the darknet describing the pandemic as a unique opportunity to entice children online and including instructions on how to access children to produce and share CSAM.^28^–^30^ The National Center for Missing and Exploited Children (NCMEC) experienced “an explosion in reporting” to their *CyberTipline* early on.^28^,^29^ In May 2020, during the first wave of shutdowns, reports to the NCMEC tipline numbered almost 1.7 million, as compared to ≈745,000 reports in May 2019.^29^

According to NCMEC, reports involving at-risk children from across the country increased by 28% from an average of ≈326,680 per week in 2019 to a weekly average of ≈418,290 reports during 2020.^28^ Reports of online enticements experienced an exponential growth of 97.5% from 19,174 total reports in 2019 to 37,872 in 2020.^28^ The dramatic rise in criminal cyber activity and the concomitant risk to children are thought to be related to increased time online while socially distancing, adult boredom, and preoccupation with sexual thoughts, and a doubling in the number of chatters on CSAM forums since the start of the COVID-19 pandemic.^30^

Survivors of trafficking in recovery, already struggling to establish themselves socially and financially, have had to endure food and housing insecurity and lack of employment opportunities during the general economic downturn. While some benefited from eviction moratoriums, many others were left homeless due to job loss and inability to pay rent. Socioeconomic stressors associated with the pandemic increased the risk of survivors being re-trafficked and of at-risk individuals being newly trafficked. The pressures for money to pay for food, housing, and other necessities may lead individuals to accept exploitative work, engage in commercial sex work, and commercially sexually exploit children.

To compound the problem, frontline health and social service organizations—and the precarious local mechanisms for referral—experienced severe disruptions of their everyday outreach and service activities. Any legal or immigration proceedings in progress prior to the start of the pandemic likely were unexpectedly suspended resulting in prolonged states of abeyance, uncertainty, and non-closure for victims and survivors. Consequently, the COVID-19 pandemic may have exacted a heavier toll on the physical, mental, emotional, and financial health of victims and survivors than is currently understood.

## ELDER ABUSE AND NEGLECT

Elder abuse and neglect is “an intentional act, or failure to act, by a caregiver or another person in a relationship involving an expectation of trust that causes or creates a risk of harm to an older adult.”^31^ Types of abuse include physical, sexual, emotional or psychological, as well as financial abuse, and neglect. Before COVID-19, an estimated one in six older persons were subject to abuse globally with one in 10 US residents ≥60 years subject to abuse. Post-COVID-19 increases in elder abuse were reported worldwide.^32^ Previously mentioned risk factors may be exacerbated in elderly populations.

There are associated risk factors of elder abuse that can be assessed and managed by medical and public health professionals such as diagnoses of mental illness, alcohol use disorder, and greater degrees of financial and emotional dependence experienced by a vulnerable elder. Risk factors vary among individuals, relationships, communities, and cultures. Identified protective factors include high levels of community cohesion and coordination of resources and services for older people. With early recognition of risk factors and implementation of protective strategies, elder abuse can be prevented.

There was an increase in mental health issues for persons of all ages in part due to implementation of mandatory public health and social measures such as physical distancing, isolation, and restrictions on movement. One study reported that elder abuse increased to one in five older people in the US during the COVID-19 pandemic.^33^ Reports include those living in long-term care facilities or other community settings, as well as those living with caregivers.

Those living away from caregivers were further isolated, with less direct access to services and a decrease in available communication methods. Older people often have less technologic access and literacy, making it difficult to navigate without in-person support. Given their social isolation, older adults have become more dependent on caregivers, risking abuser exploitation. Caregivers had their own health and safety to worry about, as well as concerns about financial and other resources needed to care for the older persons in their life, leading to increased stress and burden on all involved, and further risk of exploitation of resources such as Social Security benefits designated for older people. Increased stigma was placed on older people, as those most severely affected by COVID-19 were sometimes given priority medical resources over younger people with a greater chance of survival.

Governments and public health professionals must acknowledge that elder abuse exists. Emergency clinicians should screen all older people for possible abuse and consider risk factors and protective factors during every encounter. Especially on the ED frontline and primary care offices, healthcare professionals must be aware of local/state reporting mandates. Any suspected mistreatment should be reported according to local/state mandates (usually via Adult Protective Services).

An impactful way to prevent mistreatment is to increase social connectedness with older people and their caregivers in our communities. With persistent physical distancing, we need to try harder to stay close socially—via phone and video calls, messaging, or outside meeting—to stay connected and check in with others to reduce isolation.

## GUN VIOLENCE

The pandemic has been associated with increased firearms purchasing both by experienced owners and first-time buyers. With the start of the pandemic, a surge in US gun sales was tied to stay-at-home orders and the first wave of pandemic-related unemployment.^35^,^36^ As the year progressed and political polarization increased, people continued to arm themselves; 40 million background checks for gun purchases were recorded in 2020.^35^,^36^ Almost one-quarter of those seeking guns had not previously owned a firearm. Woman and Blacks showed the greatest increases in firearm purchasing. Historically, increases in firearm purchases have been linked with elections or restrictive policy worries. But the COVID-19 pandemic diverged from this trend and was linked to fear associated with the pandemic, lockdown, racism, elections, and the police.

Both firearm assault and DV incidents in the US increased by 8.1% in the first months following the imposition of stay-at-home orders.^18^ People at risk of DV are at high risk of being killed by a firearm with over one half of all intimate partner homicides committed with guns.^37^ In a study conducted at Level I trauma centers across Philadelphia, Abdallah et al found that intentional or violent trauma, such as firearm violence, stabbings, and assaults, significantly increased when comparing six weeks prior to and 10 weeks after implementation of stay-at-home orders; other studies reported greater increases in shootings after lockdown was lifted.^38^ Recognizing the synergistic epidemic, or syndemic, of racism, COVID-19, and firearm injury is important. Preliminary data showed Blacks were twice as likely as Whites to die from COVID-19.^38^ Blacks are also eight times more likely to be killed by a firearm than Whites.^34^ Preliminary statistics from 2020 suggest that the COVID-19 pandemic compounded racial inequities in firearm violence. In a study conducted using the Philadelphia Police Department data registry of shooting victims, researchers noted that a spike in the number of people shot per week depended on a temporal relationship to Philadelphia’s first COVID-19 lockdown.^40^

Finally, more than half of deaths from firearms occur from suicide. Although preliminary data suggests suicide deaths dropped in 2020 compared with 2019, it is anticipated that the mental health burden of the pandemic will peak later than the actual pandemic. The 1918 Spanish Flu pandemic, for example, was associated with an increase in death by suicide, suggesting the social isolation link.^41^ Firearms were shown to reduce the time period of first suicidal thoughts and attempts, as well as to significantly increase the lethality of those attempts.^42^ With increased access to firearms, numbers of first-time buyers, and feelings of social isolation, there is a high risk of future increases in suicides related to firearm injury.

While the summative effect of the COVID-10 pandemic and the gun violence pandemic, and their relationship to each other, has not yet been studied, there is cause for concern. Recognition of risk is the first step toward improvedprevention of firearm injury. Emergency clinicians are uniquely positioned to intervene as we care for vulnerable patients who may be facing DV, racial violence, or depressive symptoms. Screening of *all* ED patients may make a difference.

## COVID-19 FEAR AND NEGLECT

During the pandemic, there was a documented decrease in ED visits for medical and traumatic conditions, myocardial infarctions, stroke, and hyperglycemic crises.^43^–^46^ Forty percent of adults deferred care for fear of catching COVID-19, leading to serious morbidity and mortality.^47^ An ACEP study found that 80% of those surveyed were concerned about contracting COVID-19 from other ED patients or visitors, and 29% actively delayed or avoided seeking medical care due to these concerns.^48^ Another survey regarding non-COVID-19-related complaints found 59% were unlikely to use the ED and another 20% “didn’t know.”^49^

Emergency physicians have countless stories of patients delaying medical care due to fear of contracting disease. Many of these patients presented very late into the course of their disease, suffering unnecessary complications and potentially permanent consequences. Such instances raise the question of when does fear of disease turn into actual neglect, and complicate the assessment of abuse and neglect, particularly of dependent populations.

Patients exercise their autonomy when deciding whether to seek care due to fear of COVID-19. What if that decision were made for the patient by someone else? Are parents neglectful when choosing not to take their child to the doctor or ED for care because of fears surrounding COVID-19? Is it child neglect if parents delay seeking care for their 9-year-old with right lower quadrant abdominal pain for a week resulting in perforated appendicitis requiring percutaneous drainage, prolonged parenteral antibiotics, and delayed appendectomy? Is this reportable to CPS?

Conversely, there is the example of an elderly woman who falls with immediate hip pain and inability to walk and whose refusal of family members’ offer to take her to the ED leaves her bedbound for a week. Family bought diapers for toileting and gave her meals in bed. Finally, when the pain escalated, she agreed to present to the ED where she was diagnosed with an intertrochanteric hip fracture with associated deep vein thrombosis. Is this self-neglect or elder neglect? Nursing home staff assume a resident with fever, body aches, abdominal pain, and nausea/vomiting/diarrhea has COVID-19 during an outbreak in their facility and don’t send her to the ED for three days despite a negative COVID-19 test. She was found to have incarcerated bowel with sepsis. Was the nursing home neglectful for anchoring on COVID-19 because of their facility’s concomitant outbreak? Are these events reportable to Adult Protective Services?

Child neglect is defined as the failure of a parent or other person with responsibility for the child to provide needed food, clothing, shelter, medical care, or supervision to the degree that the child’s health, safety, and well-being are threatened with harm.^50^ Similarly, elder neglect is defined as failure by a caregiver or other person in a trust relationship to protect an elder from harm or the failure to meet needs for essential medical care, nutrition, hydration, hygiene, clothing, and basic activities of daily needs or shelter, which results in a serious risk of compromised health and/or safety relative to age, health status, and cultural norms.^31^ Self-neglect is the behavior of an elderly person that threatens his/her own health and safety.^31^

During non-pandemic times, most clinicians would believe that these questions posed above were proof of neglect. However, pandemic fears, especially during surges, made this judgment difficult. In assessing situations, several factors must be considered, including intent, expectation of trust, risk, and harm. The key question to consider is “what was the intent or intentionality of the decision?” Was delaying caremalicious or honoring the patient’s wishes? In cases of self-neglect, the competence and decision-making capacity of the patient must be considered. It is not self-neglect if the patient has competence and decision-making capacity.^31^

Classifying actions as neglect requires thought. One does not want to wrongfully accuse or, conversely, miss red flags and possibly subject the patient to more serious abuse. Emergency physicians should employ ED social workers to help with difficult cases. With regard to COVID-19 fear, local and national organizations needed to educate the public about seeking appropriate medical care and the true risk of disease transmission in healthcare settings. Patient education is key to ensure timely and appropriate medical care. With the advent of vaccination, hopefully these fears were lessened.

## LIMITATIONS

The authors who performed the literature search and review were not blinded. The true extent of pandemic-related violence remains, and will likely remain, incompletely reported and understood. As a result, the available quantitative data is limited. Anecdotal evidence suggests that pandemics require increased vigilance for signs of interpersonal abuse and violence. Moreover, and perhaps more pressing, a thorough risk-benefit analysis of universal lockdowns as a mitigation strategy must be conducted to further our understanding and ensure adequate emergency preparedness during future respiratory pandemics, given a much earlier prediction that lockdowns would be of little help and would increase violence. Finally, we did not focus on specific prevention techniques related to COVID-19, as research on this subject was sparse.

## CONCLUSION

It is evident that measures meant to help control the spread of the COVID-19 pandemic had many unintended consequences and placed people at risk for violence. The pandemic left abuse and violence victims feeling isolated with fewer options for help and decreased opportunities for recognition. Hospitals and violence prevention programs need to start planning for the next pandemic with a focus on preserving or expanding access to services, strengthening social service agency partnerships, and ensuring these agencies have access to the ED with proper PPE.
